# Experiences of inequitable care among Afghan mothers surviving near-miss morbidity in Tehran, Iran: a qualitative interview study

**DOI:** 10.1186/s12939-017-0617-8

**Published:** 2017-07-07

**Authors:** Soheila Mohammadi, Aje Carlbom, Robabeh Taheripanah, Birgitta Essén

**Affiliations:** 1 Department of Women’s and Children’s Health, International Maternal and Child Health (IMCH), Uppsala University, SE-751 85 Uppsala, Sweden; 2 Faculty of Health and Society, Malmö University, Malmö, Sweden; 3 Infertility and Reproductive Health Research Center (IRHRC), Shahid Beheshti University of Medical Sciences, Tehran, Iran

**Keywords:** Maternal near miss, Care experiences, Afghan migrants, Mistreatment, Discrimination, Iran

## Abstract

**Background:**

Providing equitable maternal care to migrants is a seriously challenging task for hosting countries. Iran, the second-most accessed country for refugees from Afghanistan, has achieved maternal health improvement. However, Afghan women with near-miss morbidity faced pre-hospital delays and disparity in maternal care at hospitals. This study explores experiences of maternal care among Afghan women surviving near-miss morbidity to increase insight into healthcare improvements for migrants.

**Methods:**

A qualitative study was conducted at university hospitals in Tehran, from April 2013 to May 2014. A total of 11 Afghan women and 4 husbands were interviewed when women recovered from near-miss morbidity that occurred around the childbirth period. Mothers were identified prospectively using the WHO maternal near-miss approach. Thematic analysis was used along with a data-driven approach to organize data guided by the ‘three delays model’ theoretical framework.

**Results:**

Mistreatment in the form of discrimination and insufficient medical attention were key experiences. Participants commonly perceived poor women–professional communication and delays in recognizing obstetric complications despite repeated care-seeking. Financial constraints, costly care, lack of health insurance, and low literacy were experienced barriers to accessing care to a lesser extent. Non-somatic consequences of near-miss morbidity affected mothers and families for extended periods.

**Conclusions:**

Near-miss survivors’ experiences provided remarkable insights into maternal care of Afghans in Iran. The challenge for the health system and professionals is to provide equitable care with dignity and improve communication skills with caring attitudes toward ethnic minorities. Antenatal visits provide the best and most appropriate opportunities to tackle health illiteracy in Afghan women.

## Background

Millions of women of childbearing age have fled conflict and crossed national borders, making equitable maternal care provision a serious challenge for hosting countries [1, 2]. Afghanistan is ranked second in the world in terms of its number of refugees, with massive displacement of its population due to domestic upheaval, a long-term war, widespread violence, and insecurity [3, 4]. Women in Afghanistan are affected by a low adult literacy level (31%) which, along with tradition and religious extremism, marginalizes women and inadvertently leads to discrimination in various aspects of their lives in this country [5], particularly in relation to maternal health. For example, the majority of Afghan girls face forced marriage before the age of 16 years, the fertility rate is 4.9 children per woman, and the Maternal Mortality Ratio (MMR) of 396/100,000 live births in this country is one of the highest in the world [5, 6]. Home births are popular and skilled health personnel only attend 36% of childbirths [7].

After Pakistan, Iran is the second-most accessed hosting country for Afghan refugees, and they share similar languages (Farsi and Dari) and the Shia Islam religion [4]. The youth literacy rate for females (98%), antenatal care attendance (94%), institutional birth rate (95%), total fertility rate (1.9), and MMR (25/100,000 live births) are all indicative of Iran’s achievements in improving women’s health [6, 8]. However, our recent study of maternal near-miss (MNM) morbidity in Tehran shows that women are subjected to pre-hospital delays and Afghan women face disparity in obstetric care quality at hospital [9, 10]. MNM is defined as a woman who, while pregnant, during childbirth, or within six weeks postnatal, nearly dies but survives critical conditions [11]. MNM is an obstetric indicator for evaluating maternal care quality. As women who experience near-miss morbidity survive, their personal accounts of delays and care experiences can benefit strategies for maternal care improvements [12].

This study aimed at exploring the experiences of maternal care among Afghan near-miss survivors in Tehran to increase insight into strategies for reducing delays and providing optimal and equitable care for migrants.

## Method

### Study setting

Tehran, with a population of over 12 million, hosts one million Afghans, of whom only one-third possess legal documents [13, 14]. A high level of availability of healthcare services and the overuse of cesarean section (CS) characterize this setting [9]. Antenatal visits are included in primary healthcare and are provided free of charge at public health centers, even for migrants. Inpatient care is provided through 140 public and private hospitals in this metropolis. Until 2015, the costs of receiving such care at public hospitals were covered by health insurance for Iranians who worked or who could afford to pay the premiums, while uninsured natives and migrants had to pay all of the care costs [15, 16]. After recent health system reforms, a new public health scheme covers inpatient care costs for all Iranians and registered migrants [16]. Our study sites comprised one secondary and two tertiary university hospitals in Tehran.

### Study design and population

A qualitative interview study was conducted as part of a larger project investigating MNM and obstetric care quality at university hospitals in Tehran. During the first phase of the project, 82 MNM women (60 Iranians and 22 Afghans) were prospectively identified during daily morning reports using the WHO near-miss approach [9]. The quality of the obstetric care provided to 76 of these women was assessed within an audit study as the second phase of the larger project and the results have been presented in detail elsewhere [10]. During the present study, semi-structured interviews were conducted with 11 Afghan MNM women and 4 men. To gain an in-depth understanding of the factors that could affect Afghans’ maternal care, a purposive sample of Afghan MNM survivors was employed, with possible variations in terms of age, parity, education level, and length of residency in Iran. We recruited 11 near-miss survivors while they recovered from critical events but who were either still in hospital or who had returned, up to six months after hospital discharge. Four Afghan men who accompanied their wives when they returned to the hospital were also interviewed. It was impractical to conduct face-to-face interviews with three of the women; therefore, telephone interviews were performed instead. The main researcher, an obstetrician who had previously worked at these hospitals, held the interviews in Farsi. Firstly, she introduced herself as an obstetrician and researcher who had no clinical or managerial responsibility at those hospitals and explained the objectives of the study to the participants. Then, questions about the women’s socio-demographic backgrounds and the length of their residency in Iran were asked. The interviewer did not ask participants to declare their legal status due to the sensitivity of the issue and the potential effect on the women’s willingness to participate in the meetings. The interview themes included experiences of antenatal visits, childbirth and postnatal care, the information they received, the way they reached a decision about where to give birth, affordability of maternity services, and perceptions of care quality. The interviews were tape-recorded and the original transcripts were developed in Farsi by a transcriber after each interview and were validated by the main researcher. Information from one interviewee was presented to and commented on by subsequent interviewees to increase credibility by way of member checking. The interviews lasted between approximately 30 and 80 min each. We recruited new participants until no new information was retrieved and theoretical saturation was obtained. Lastly, the data were translated to English with the help of two independent translators, and the co-authors worked closely to interpret and organize the data.

### Analysis

Thematic analysis, as described by Braun and Clarke, was used to analyse our interview data [17]. The analysis commenced by performing multiple readings of transcripts and listening to the tapes. We used a data-driven approach to develop codes from the interview data itself rather than searching deductively for the interview themes. An explicit level of analysis was employed and the list of codes was generated directly from what participants said while critically reflecting on the interview data [17]. The codes were then mapped to identify patterns and themes in later stages of the analysis.

We presented the experiences of delays in achieving maternal care in light of the ‘three delays model’ developed by Thaddeus and Maine (1994) [18]. This model is based on three phases of delays: Phase I, delays in decision-making and care-seeking for reasons of cost, inadequate knowledge, inadequate perception of illness severity; Phase II, obstacles and delays in accessing medical facilities for reasons of distance and transportation (both phases at pre-hospital level); and Phase III, delays in receiving appropriate care at hospital for reasons of shortages in professional resources, medical supplies, and suboptimal care.

## Results

### Study participants

All participants were married Muslims who had lived in Iran for between 3 and 20 years. They were aged between 18 and 31 years and almost half of them had more than three children. Except one couple, all had low income and half of them were illiterate. All interviewees were uninsured; however, they attended antenatal visits and underwent blood tests and several ultrasound examinations during pregnancy, incurring high costs. Six mothers arrived at hospital in a moribund condition or they developed near-miss events within six hours of hospital admission. The findings are grouped into three main themes; ‘care experiences and pre-hospital delays’, ‘maternal experiences and delays at hospital, and ‘MNM consequences’, and are described below.

### Care experiences and pre-hospital delays

Poor women–professional communication was a common experience within participants’ narratives. Several interviewees claimed that obstetric professionals were busy and had no time for careful interaction. Participants often thought that the obstetric professionals were unqualified to diagnose the illness. As they described, doctors did not recognize their problems in a timely manner or they made misdiagnoses, despite our participants having sought care, sometimes repeatedly.

Afghan migrants live in city boundaries where unqualified doctors and midwives work. My wife felt ill and had headache in the last week of her pregnancy. I took her to the nearest clinic two–three times but they (doctors) didn’t recognize her problem. Neither did they adequately talk with us. They trifled with Afghanis. (Husband of participant 3)

One woman developed postpartum sepsis and underwent obstetric hysterectomy two weeks after her childbirth. She had firstly been taken to a clinic and received syrup for an allergic reaction before she became worse and was taken to the hospital. She reported:

I have often headache and feel sick, but I don’t want to seek care any more. I do know that doctors are unable to identify my problem. (Participant 2)

Women generally had received inadequate information about complications that could potentially affect a pregnant mother and her baby. The only memories that all participants could recall from the antenatal visits was that their blood pressure was taken, the fetal heart rate was listened to, and they were given iron pills. Several mothers could not believe what had happened to them as they recalled being told they had no problems and everything was fine with the baby. According to our participants, all Afghan women attended midwifery consultations for antenatal care but rarely did they receive proper counseling. These participants thought obstetric professionals did not really engage with the care provision nor did they show a caring attitude towards them. An educated woman who developed severe preeclampsia late in her second pregnancy explained:

I asked my doctor some questions during visits, but neither did she answer my questions nor explain any other thing for me. She might think I’m Afghani and didn’t understand. Therefore, I didn’t know which dangers might arise while being pregnant, but who cares for us? (Participant 3)

Her husband added:

All Afghan women attend public health centers for pregnancy check-up. However, rarely did midwives adequately consult them. Therefore, women are still faced with problems. Women would definitely care and seek health services had they been fully aware of the potential dangers to their health and the health of their babies. (Husband of participant 3)

Participants were generally confronted with intertwined factors during pregnancy and childbirth. Economic constraint was commonly reported as a barrier to the timely seeking and accessing of care services. Financial problems were particularly highlighted when participants described costly care services and their lack of health insurance. Although several interviewees mentioned that costly services were a burden, they did seek care and underwent the recommended tests and ultrasound examinations. An illegal refugee with previous CS who lived in poverty stayed at home when uterine contractions had started and was taken to the hospital when the uterus ruptured and the baby died in her womb. She reported that the cost of a hospital birth was too much when they could barely afford food expenses. However, she added that the outcome could have been different had the midwife informed her that the baby might die if she did not deliver in hospital.

Having a low education level was also highlighted within the narratives. Our participants thought illiterate women could barely understand what professionals advised them during the visits. They thought these women might be embarrassed to ask irrelevant and inappropriate questions, thus, they preferred to stay quiet.

Women occasionally mentioned that the decisions their parents-in-law made were a barrier to timely care-seeking. A woman with epilepsy was taken to the hospital one week after having had a home birth while she was in a septic condition. She heard her mother-in-law say that there was no money to take her to the hospital despite her having had several seizures at home. Another woman claimed that the cost of care mainly affected the decisions a mother-in-law made. She believed that all Afghans, including mothers-in-law, would prefer institutional birth had it been provided free of charge.

### Maternal experiences and delays at hospital

Our participants mainly experienced mistreatment in the form of being subject to discrimination and insufficient medical attention in the course of receiving maternal care. A mother to five children developed severe postpartum hemorrhage 10 days after elective repeat CS and underwent obstetric hysterectomy. Subsequently, she developed severe complications that required re-admissions, re-operations, and prolonged hospitalization. According to her husband, not only did she experience these critical illness episodes one after another, but also, she suffered from being subjected to discrimination and ignorance. He said:

Afghanis usually don’t get any attention until they came to dying. My wife is illiterate and embarrassed to ask any question. We never get the right help. I took my wife to her doctor’s private clinic several times but the doctor didn’t take her complaints seriously. My wife had terrible pain and I didn’t know what to do. It was horrible. My wife and my family have suffered a lot. My wife underwent four surgeries, yet feels sick, but doesn’t want to seek help anymore. She feels afraid to go to the doctors. (Husband of participant 4)

Another mother gave birth by CS and afterward developed intra-abdominal bleeding. She underwent re-operation and was then subjected to ureter injury accompanied by long-lasting follow-up care. She contended that the CS was not an informed choice and her husband had inadequate funds to pay the entire cost upon her discharge from hospital. They experienced serious discrimination and disrespect while paying the hospital bill.

At the accounting department, they said to my husband that we shouldn’t ask why we have to pay so much, but be grateful for being admitted at hospital in spite of being Afghani. (Participant 6)

She further added that her responsible doctor helped them later on with the payments, understanding that the bill had become unaffordable.

Another participant thought that nurses treated Afghans and Iranians differently. She observed that when Iranians questioned something in relation to care services, nurses did not react defensively, but if Afghans did so, nurses shouted and cursed them. While participants often claimed that hospitals did not admit unregistered Afghans, it was occasionally cited that hospitals did not admit Afghans, regardless of whether they had a residence permit.

Interviewees even mentioned experiences of being discriminated against in society. For instance, an educated Afghan man reported that even those who had legal permission to live in Iran had no health insurance despite having lived and worked there for many years. One man, who had lived in Iran for 20 years, spoke Farsi, looked Iranian, and had worked for one business for many years, claimed that his employer had not paid for his insurance because of his Afghan nationality. Another man, who had lived in Iran for 12 years, expressed how he felt like an ‘outsider’ in Iranian society, as follows:

After all, we know that we are strangers. We have nothing here in Iran. Everything from job, income, pension, to facilities, services, and insurance belongs to Iranians. (Husband of participant 2)

Participants commonly said that obstetric professionals paid insufficient attention to their complaints and delayed treating disorders. They thought their voices were not heard at the hospital. Several mothers related the near-miss events they developed to the delays they had experienced. They thought that the burden they faced could have been prevented had obstetric professionals paid sufficient attention to them. A mother with intra-abdominal bleeding after CS explained:

I felt very ill after operation. My aunt said to nurses several times that I felt ill; they told her to give me something to drink. They didn’t care; they didn’t take me seriously. Finally, when my aunt helped me to sit I fainted. (Participant 7)

Verbal abuse was also cited. One woman thought that obstetric professionals behaved as though Afghans were stupid. She recalled the popular sentence that Afghans heard: ‘You are Afghanis; if your baby dies, you’ll come back next year with another’. One woman recalled memories of her first childbirth at a maternity center, where she heard midwives say that they were unable to put up with Afghans any longer.

Participants generally mentioned that they were not informed about what had happened to them at the hospital. They thought doctors did not care to explain anything to them. Mothers had inadequate insights about the complications they developed and the reasons they were delivered by CS even after their successful recovery and discharge from the hospital. These kinds of experiences broke the necessary trust between patients and professionals and appeared to affect care-seeking in the future.

A few women, however, were satisfied with the care services they received and expressed their thankfulness to God for being alive after experiencing all of those complications.

We found several narratives suggesting that women received good support from their Afghan husbands. One woman said:

I had three home births, but my husband told me that I should give birth at hospital this time because it is more convenient and they have everything to take care of the baby and me if something happens. (Participant 8)

Another woman cited:

My husband said even if he had to beg for the money in the streets to pay the care costs for me he would, but I wouldn’t want to spend food expenses to pay the hospital bill. (Participant 11)

One of the men claimed that all the Afghan men he knew worked hard to earn money and take their wives to the hospital for childbirth. Another man expressed how he thought that a mother is the core of the family and every effort should be made to keep her healthy.

### Maternal near-miss consequences

Within the interview data, some narratives were found that related to the long-lasting consequences of near-miss morbidity. Women reported that their husbands were unable to work regularly when these women were hospitalized. Men should undertake some healthcare tasks, such as buying the prescribed medications for substitution, and taking blood samples to the laboratories outside the hospitals.

According to the narratives, the cost of care was incredibly high and burdened our participants with huge debt and with the stress of paying off the creditors who repeatedly asked for the money. A young woman said that the relationship she had with her husband was negatively affected by the near-miss events. She further explained that her husband had experienced numerous challenges during the period of her illness, from extensive stress of his wife being in critical condition and requiring long-term hospitalization, to not being able to work to ease the burden of serious debt. Therefore, he could not put up with the baby and with her complaints about pains at home any longer.

One woman with epilepsy who survived sepsis reported that her parents-in-law blamed her for the illness and the death of her baby. They pushed her husband to divorce her. Another woman who lost her uterus believed that she could no longer perform her important duty to give birth. This feeling made her depressed and affected her ability to function as mother to three children at home.

## Discussion

This study explored various experiences of maternal care among Afghan near-miss survivors at university hospitals in Tehran. Much of the first-hand information that participants shared highlights the importance of obstetric professionals’ knowledge and the quality of their communication skills to provide positive experiences of care. In particular, we detected experience of discrimination on the grounds of nationality in the course of receiving maternal care. Moreover, an inequitable healthcare policy in terms of insurance coverage was cited as a major barrier to accessing care. Poverty, and having a low education level, to a lesser extent, were contributing factors to delayed care.

### Pre-hospital delays

Our participants actively attended antenatal consultations and most made an informed choice to have a hospital birth despite their familiarity with home birth and their low family income. Although all participants were uninsured and all but one family had financial limitations, and despite variations in their legal statuses, only two women considered that their late arrival to hospital was related to poverty and lack of a residence permit. Other participants who had MNM upon arrival claimed that they had made repeated visits to obstetric professionals before developing near-miss events. Therefore, well-known factors contributing to delays in seeking and accessing care, such as poverty, illiteracy, and being an undocumented refugee, might, to a lesser degree, explain the pre-hospital delays in our setting [1, 12]. Near-miss survivors perceived that poor communication, ineffective consultation, and lacking a caring attitude toward Afghans often contributed to the delays they faced and were related to developing a critical condition. Participants thought their health was not the obstetric professionals’ real concern; therefore, repeated care-seeking did not result in accurate diagnosis and careful guidance. This finding agrees with a recent observation suggesting the inferiority of Afghans’ position among health workers in Iran [19]. In a similar manner, migrant women in high-income settings commonly express a correlation between caregivers paying insufficient attention to their complaints and the development of severe morbidity [20, 21]. Inadequate antenatal consultation can impede the timely recognizing and managing of complications and fail in providing useful information for women [22]. As caregivers’ behavior is a crucial component for achieving desirable outcomes, suboptimal practice can contribute to pre-hospital delays, even when care services are available [23].

Our participants frequently referred to the burden of the care costs and the lack of access to health insurance for migrants. Due to international sanctions and financial limitations, Iran has limited resources for covering the required care and many people incur high out-of-pocket expenses (up to 55%) [24]. As previous publications show, the lack of insurance coverage for hospital care before the 2015 reform contributed to an elevated risk of MNM among Afghan women and contradicts the basic right of disadvantaged mothers to access emergency care [9, 16].

### Disparity in maternal care

Many interviewees felt that they had been subjected to discrimination at hospitals. Inequitable health policies and degrading treatment in combination with socioeconomic disadvantages appeared to shape a perception of dissociation from the Iranian health system and society among our participants. Women are subjected to discrimination based on ethnicity, race, age, socioeconomic status, and religion while giving birth at health facilities worldwide [25]. The perception of inequity and disrespectful care by Afghan refugees in Mashhad, Iran has been shown recently [19]. In Bolivia, socioeconomically disadvantaged women perceive themselves as being separated from “others”, a perception that reinforces their dissociation from the health system [26]. Women face overt discrimination based on gender in Afghanistan and their narratives in the present study shed light on a covert discrimination based on their nationality in Iran. National guidelines for obstetrics emphasize the provision of good quality and respectful maternal care [27]. Moreover, the patients’ bill of rights, enacted in 2002, clearly states that all individuals have the right to appropriate care with dignity and respect, regardless of personal background [28]. Beyond the guidelines, human equity is fundamental in Islam: there is no superiority or inferiority of any people based on gender or ethnicity (Quran 49:13). God orders justice and forbids any form of unfair treatment (Quran 16:90). However, our participants commonly expressed the denial of their basic rights while receiving obstetric care. They would obviously want to know what had happened to them during the period of their critical illness, and this is a right that is even included in the WHO vision of quality for maternal care [29]. According to the accepted code of ethics in obstetrics and gynecology, professionals should fully inform mothers of any potential risks and management alternatives and give women the opportunity to ask questions and obtain their input and consent within the decision-making process [30]. However, the literature shows how women in various settings complain about receiving inadequate information as well as ineffective communication during the maternal healthcare interchange [31]. The shortage of care professionals, inadequately qualified staff, and a careless attitude among professionals are suggested to be the leading causes of ineffective consultations and poor practice [25, 32].

**Fig. 1:**
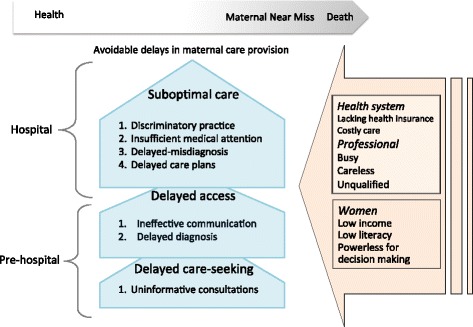
Conceptual model, inspired from Thaddeus and Maine [19], mapping Afghans’ experiences of care to propose new hypotheses about delays in maternal care in Tehran, Iran

We applied our findings within the ‘three delays model’ and generated new hypotheses about the avoidable delays that Afghan women faced in a setting with high availability of obstetric services [18]. In the spectrum between health and death, MNM lies near death (Fig. 1). Health system, care professionals, and women themselves influence the maternal health condition during pregnancy and childbirth [33]. Our participants’ accounts proposed a hypothesis that uninformative consultations, ineffective communication, and delayed diagnosis could contribute to pre-hospital delays in a metropolis. The second hypothesis generated from our results is that discriminatory practices and the insufficient medical attention given to Afghan women might underpin the disparity in maternal care quality and inequitable outcomes for migrants. We acknowledge that individual factors could intertwine with health system functions and professional performance and thus affect the achievement of timely care. Further research is required to test the new hypotheses created in the present study.

In line with other publications, we found that women and families shouldered a serious burden of consequences after near-miss morbidity [34]. The support provided by Afghan husbands was a contradictory finding when considering the violence against women and gender discrimination that is presumed to take place in Afghanistan [7, 35]. Further study is required to elaborate on the behavioral changes among Afghan men after migration.

To the best of our knowledge, this study was the first attempt to investigate maternal care experiences of Afghan women in Iran. The interview data gave us a profound understanding about how delays in achieving optimal care shaped maternal experiences of migrants at pre-hospital and hospital levels. The heterogeneity of the participants assisted in obtaining general insight, while the presence of Afghan men during the interviews might have affected the women’s ability to talk about intra-household experiences comfortably. Using a qualitative method alongside our larger MNM study that thoroughly assessed the quality of care provision showed us the intersection between socioeconomic, cultural, and behavioral measures and care outcomes. The position of the main researcher as an outsider, yet an obstetrician who knew the field and language, appeared to provide adequate levels of trust during the interviews. Nevertheless, employing an interviewer who was not an obstetrician or who was unaware of the care quality provided to the participants could have obtained other responses or they may have analyzed the data differently. Moreover, care experiences found among Afghan participants could have been similar to that of natives, but further study is needed to explore such experiences among Iranians.

The participants were selected based on a hospital study; therefore, there is the potential that we could have missed the perceptions of those women who were unable to reach hospitals. Holding interviews in hospital might be a source of bias in our results because women had to recall the experience of the critical illness or they may have felt uncomfortable in talking freely about their caregivers [12]. We are aware that including the voices of obstetric professionals might also have added to the whole understanding.

## Conclusion

This study highlights various factors that contributed to delays and disparity in the maternal care provided to Afghan migrants. Our results explored the negative experiences of being subjected to discriminatory care and receiving degrading treatment in Tehran. Therefore, it is essential for obstetric professionals to be aware of how mothers experience the care they provide. Moreover, adopting a rights-based approach in training and in monitoring maternity services may ensure effective, respectful, and equitable care. Migrants affected by socioeconomic and humanitarian challenges and the special needs of this minor population, such as health literacy, should be given additional attention within antenatal consultations. To improve migrants’ maternal outcomes, policy makers should consider barriers to achieving equitable care and should include maternal voices when developing and structuring health programs.

## Abbreviations

CS: Cesarean section
MMR: Maternal mortality ratio
MNM: Maternal near miss WHO World Health Organization
